# Evaluation of Boarding Methods Adapted for Social Distancing When Using Apron Buses

**DOI:** 10.1109/ACCESS.2020.3015736

**Published:** 2020-08-11

**Authors:** R. John Milne, Camelia Delcea, Liviu-Adrian Cotfas, Corina Ioanăş

**Affiliations:** 1 David D. Reh School of BusinessClarkson University 6704 Potsdam NY 13699 USA; 2 Department of Economic Informatics and CyberneticsBucharest University of Economic Studies 125536 010552 Bucharest Romania; 3 Department of Accounting and AuditBucharest University of Economic Studies 125536 010552 Bucharest Romania

**Keywords:** Airplane boarding, social distancing, COVID-19, SARS-CoV-2, apron buses, agent-based modeling, NetLogo

## Abstract

Social distancing reduces the risk of people becoming infected with the novel coronavirus (SARS-CoV-2). When passengers are transported from an airport terminal to an airplane using apron buses, safe social distancing during pandemic times reduces the capacity of the apron buses and has led to the practice of airlines keeping the middle seats of the airplanes unoccupied. This article adapts classical boarding methods so that they may be used with social distancing and apron buses. We conduct stochastic simulation experiments to assess nine adaptations of boarding methods according to four performance metrics. Three of the metrics are related to the risk of the virus spreading to passengers during boarding. The fourth metric is the time to complete boarding of the two-door airplane when apron bus transport passengers to the airplane. Our experiments assume that passengers advancing to their airplane seats are separated by an aisle social distance of 1 m or 2 m. Numerical results indicate that the three variations (adaptations) of the Reverse pyramid method are the best candidates for airlines to consider in this socially distanced context. The particular adaptation to use depends on an airline’s relative preference for having short boarding times versus a reduced risk of later boarding passengers passing (and thereby possibly infecting) previously seated window seat passengers. If an airline considers the latter risk to be unimportant, then the Reverse pyramid – Spread method would be the best choice because it provides the fastest time to board the airplane and is tied for the best values for the other two health risk measures.

## I. Introduction

Apron buses are commonly used to transport passengers between the airport terminal and the airplane. European airports such as Amsterdam Schipol, Madrid, Munich, Pisa, London, Luton, Frankfurt, Henry Coandă Bucharest Otopeni, Salzburg, Stuttgart, Kharkiv, use apron buses to transport passengers for some flights as well as the classical jet bridges for other flights [1]. The practice of using apron buses is common also at small airports in Europe, for example, to avoid passengers needing to walk directly from the gate to the airplane [2].

Some airlines have adapted their boarding passes to help their passengers choose the correct airplane door for boarding once they have left the apron bus [3]. The scientific literature proposes new methods for segregating passengers into groups prior to apron bus boarding to reduce the airplane boarding time [1]–[6].

Due to the novel coronavirus (SARS-CoV-2) [7]–[9], the traveling capacity of an apron bus is considerably reduced because of the need for a social distance surrounding the radius of each passenger within the apron bus.

A report released by European Union Aviation Safety Agency (EASA) in May 2020 states that, “Where buses are used in the boarding process, an increased quantity should be considered in order to accommodate for physical distancing inside them” [10], p. 12.

Recently, COBUS Industries GmbH released [11] on their Twitter and Facebook pages two diagrams of their most common apron buses models, COBUS 2700s and COBUS 3000, along with their capacities when social distance is considered. With social distancing, they suggest that the COBUS 2700s model—that normally accommodates 77 passengers—can now carry only 10 passengers safely according to their schematic. Similarly, for the Cobus 3000 model—that normally has a capacity of 110 passengers—with their proposed social distancing, it can now accommodate a capacity of only 17 passengers.

In consideration of the airplane passengers’ safety during COVID-19 outbreak, a series of distancing norms have been issued by air transportation associations and airlines with regard to social distancing when passengers are inside the airplane. The International Air Transport Association (IATA) Medical Advisory Group recommends: a minimum distance among passengers that can range from one to two meters, a limit on the number of passengers passing each other in the airplane’s aisle, a limit on the carry-on luggage, leaving every other seat empty, leaving empty seats in the jump seats region, and sequential boarding beginning with passengers who have seats in the rear of the airplane and window seats [12]. According to a European Union Aviation Safety Agency (EASA) report, the agency airline operators should ensure that while inside the airplane, a physical distance among passengers is ensured [10].

Airlines have already considered the social distancing of their passengers. Delta Air Lines [13] and GoAir [14] have adopted a back-to-front boarding approach. Southwest Airlines [15] uses the 10-passengers group boarding approach. EasyJet [16] trialed individual boarding based on seat numbers. Alaska Airlines and Wizz Air [17] mooted keeping middle seats unoccupied.

With the current air transport conditions generated by the COVID-19 pandemic, not only is the time to complete boarding of the airplane important, but it is also necessary to offer passengers a safe travelling environment [18], [19].

**FIGURE 1.**
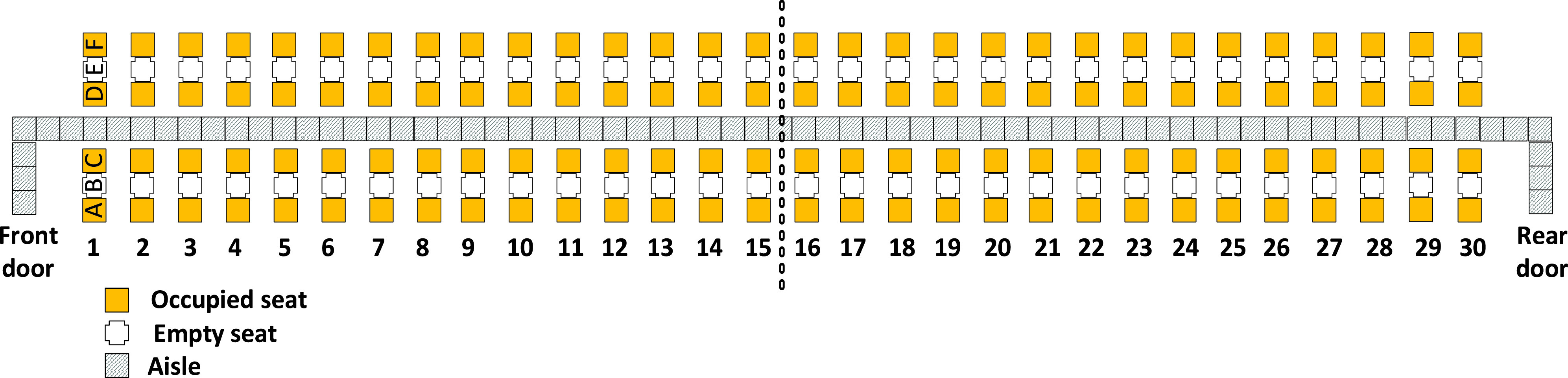
Airplane configuration modeled in this article.

The aim of this article is to adapt classical boarding methods for the social distancing conditions stemming from the pandemic and to evaluate them when apron buses are used. In particular, we propose leaving the airplane’s middle seats unoccupied and for boarding passengers walking or standing in the aisle to be separated from each other by a minimum social distance of 1 – 2 m. We refer to this minimum social distance as the *aisle distance*. We assume each apron bus has a capacity of 12 passengers; this results in 10 apron bus trips for the 120 occupied seats on the two-door airplane (that had been designed for 180 passengers before COVID-19) as depicted in Figure 1.

Following recent research [18], three health metrics (aisle risk, window risk and number of seat interferences) and one operational metric (airplane boarding time) are used for evaluating the performance of the proposed boarding methods. The health metrics reflect the risks of virus spread by contagious passengers through air and surfaces, while accounting for aisle social distancing between adjacent passengers walking down the aisle towards their seats.

The remainder of the paper is organized as follows: section 2 provides a literature review, with a focus on the best-performing airplane boarding methods when both doors of the airplane are used for passenger boarding. Section 3 describes nine adaptations of these methods for the social distancing conditions. Section 4 presents the metrics and scenarios used for the evaluation of the boarding methods, while Section 5 describes the agent-based model implemented in NetLogo 6.1.1 for conducting stochastic simulation experiments. Section 6 provides the results of the numerical simulations and discusses the performances of the tested methods considering health and operational metrics. The paper closes with a conclusion section and references. The paper is accompanied by supplementary materials in the form of videos containing simulations of all nine methods tested for 1 m and 2 m aisle social distancing.

## II. Literature Review of Boarding Methods

The process for boarding an airplane has been one of the most discussed topics in the scientific literature pertaining to the reduction of the turn time and the corresponding costs associated with the airplane turnaround process [20], [21].

Most of these studies have focus on developing or testing airplane boarding methods under various conditions [6]. As a result, different assumptions have been made related to: airplane characteristics - in terms of number of seat rows, the presence or absence of the business or first class passengers, the number of aisles [22]–[26], the degree of airplane occupancy – ranging, in general, between 60% and 100% [5], [22], [23], [27]–[31]; passenger movement assumptions [32], [33]; the presence of carry-on hand luggage – including situations with no luggage, one luggage (small/large), two (small/one small and one large) or three pieces of luggage [22], [28], [30], [32], [34]–[36]; the process of seat selection – open seating or assigned seats [24], [33]; the occurrence of boarding interferences—characterized by blocked passengers in the aisle due to either passengers stowing luggage or passengers leaving their seats to make space for a later boarding passenger [25], [29], [34], [37]–[40]; groups of passengers traveling together [6], [34], [41]; the presence of disturbances—such as the arrival of late passengers [24], etc.

Based on these assumptions, a series of boarding methods have been created, most of them featuring the use of a single airplane door for boarding (either the front or the rear door) [21], [22], [25], [27], [30], [32], [34], [42]–[44]. Depending on the means of passengers transport between the airport terminal and the airplane, different methods have been proposed when the passengers are boarded using one or two jet bridges [21], [22], [25], [27], [30], [32], [34], [35], [38], [42]–[45] or two apron buses [1]–[5]. Because it is common for passengers to board an airplane via a jet bridge that connects the terminal to one of an airplane’s doors, much of the research literature focuses on one-door boarding, though two-door boarding with apron buses has received increasing attention.

An important part of the scientific literature is dedicated to extracting data from field trials to use it for testing and comparing the developed airplane boarding methods. The field trials have featured small-scaled trials with participants ranging between 36 and 600 persons [36], [39], [46], [47].

Because we focus on methods for passengers boarding through both doors of the airplane with social distancing norms imposed by the COVID-19, in the following, we discuss the boarding methods of previous researchers that provide fast boarding times (or are popular) when both doors of the airplane are used.

Three main methods have been considered for boarding the airplanes while using the front and the rear door: Back-to-front, Outside-in (WilMA) and Reverse pyramid [28], [43], [47]–[49]. All three methods belong to the “by group” methods category as their rules primarily impose the creation of several groups of passengers based on their assigned seats and boarding them following a particular sequence. Additionally, Schultz [49] considers Random boarding, Back-to-front mix (a Back-to-front variation, sometimes called “optimized block”) and Steffen (called “individual boarding”) and compares them in a one-door boarding scenario. TABLE 1 presents concise descriptions of the six boarding methods [28], [43], [47]–[50].

**TABLE 1:** Summary of the Considered Boarding Methods Rules

Boarding method	Short description
Random	With Random boarding, the passengers arrive inside the airplane in no particular order.
Back-to-front	This method imposes the formation of several groups of passengers based on their assigned seats in the airplane. The number of the groups can vary. Often, five equal-size groups of passengers are formed. When the boarding process starts, the group having seats in the rear rows (back fifth) of the airplane is called to board. The last group to board has seats in the front rows (front fifth) of the airplane.
Back-to-front mix	This boarding strategy is a variation of the Back-to-front boarding method in which the airplane is divided into six equal-size groups. The groups are formed and numbered consecutively starting with passengers seated in the rear sixth of the airplane (group 1) and concluding with passengers sitting in the front sixth of the airplane (group 6). According to this method, the group numbers board in the sequence: 1, 3, 5, 2, 4, 6. Thus when group 2 boards, it takes seats between those of groups 1 and 3, and when group 4 boards it takes seats between those of groups 3 and 5. Variations in this method are possible by considering a different number of groups.
WilMA	Also known as “outside-in” method, with WilMA, the passengers are divided into three groups based on the seat positions: near the window, in the middle of the row, or near the aisle. The first group called to board has window seats, the second group has middle seats, and the final group to board contains those passengers having the seats near the aisle. Within each group, the passengers board in a random sequence.
Reverse pyramid	This method derives from the boarding rules of the Back-to-front and WilMA methods. A series of variations are possible for this method. As a result, the number of passenger groups can vary between 5 and 10. In each Reverse pyramid variation, the groups board following a diagonal scheme starting from the rear of the airplane with a group composed mostly (or solely) of window seat passengers and concluding with a group of passengers mostly (or solely) seated in the front of the airplane near the aisle.
Steffen	According to this method, each passenger is called individually for boarding. The first passenger to board has the seat located in the last row near the window. The second passenger to board has the window seat two rows in front of the first passenger and on the same side of the aisle. The scheme continues until all the seats located in even rows (for an airplane with even rows), near the window, on one side of the aisle are occupied. The scheme continues then on the other side of the aisle (in even rows) starting from the rear of the airplane. Then, the seats located near the window in odd rows are called to board one by one starting from the rear of the airplane. The scheme continues until the final passenger boards who has an aisle seat in the first row of the airplane.

In terms of time boarding reduction, Marelli *et al.* [47] estimated a 5 minute reduction in boarding time when two doors are used instead of one. Even though the authors do not mention the baseline boarding method, the authors acknowledge that the best-performing method when two-doors are used is WilMA. Nyquist and McFadden [43] state that the non-traditional strategies (referring to WilMA or reverse pyramid) used for boarding the passengers through two doors can save 51% to 90% of the costs generated annually versus the use of a traditional boarding method with one door. Schultz [49] states that the use of a two-door boarding approach improves the boarding time by up to 41.5% when compared to the random boarding methods used with a single door. Some of the best-performing methods according to the author are: Steffen, followed by reverse pyramid, and WilMA.

## III. Boarding Methods for Apron Buses With Social Distancing

In this section, we present nine methods for boarding an airplane with apron buses and social distancing. These methods are adaptations of classical boarding methods.

Each of the methods discussed in this section divides the passengers into 10 groups, corresponding to the 10 apron bus trips, each group containing 12 passengers. Of the 12 passengers on a bus, 6 are assigned to enter the airplane through its front door and the other 6 are assigned to enter the airplane through its rear door. We assume the passengers within each of these smaller groups of 6 passengers, board the airplane in a random sequence after leaving the apron bus.

Because of social distancing between seated passengers, the middle seat has been kept empty as pictured in Figure 1.

The boarding scheme of each method is symmetrical with respect to the middle of the airplane which is designated by dashed vertical line between rows 15 and 16 in Figure 1. Passengers entering the front door of the airplane proceed to their seats in rows 1 to 15 of the airplane, and those entering the rear door of the airplane proceed to their seats in rows 16 to 30 of the airplane.

With the Random boarding method, passengers are assigned to each of the ten apron bus trips at random. Aside from the airplane’s middle seating being open and more bus trips than usual, there is no adaptation needed for Random boarding. For the other classical boarding methods, more adaptations are required for social distancing. In the first subsection below, we discuss adaptations of the Back-to-front and Back-to-front mix methods. Then we discuss three adaptations of WilMA. Finally, we discuss three adaptations of the Reverse Pyramid method.

### A. Adaptions of Back-to-Front

**FIGURE 2.**
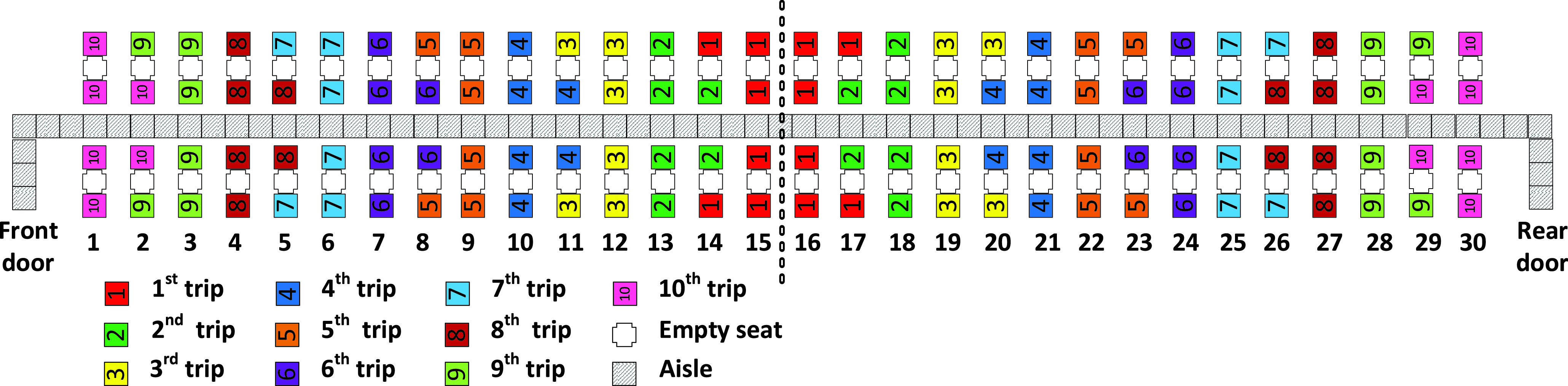
Adapted Back-to-front Method.

In the Adapted Back-to-front method, the first group of passengers (those boarding the first apron bus trip) includes all the passengers having seats in rows 15 and 16 of the airplane and the window seat passengers of rows 14 and 17 as depicted in Figure 2. The second bus trip contains the aisle seat passengers in rows 14 and 17 and all passengers with seats in rows 13 and 18. The method proceeds in this pattern until the final ($10^{\mathrm {th}}$) bus trip contains passengers sitting in rows 1 and 30 of the airplane and the aisle seat passengers sitting in rows 2 and 29.

Observe that in this adaptation of the Back-to-front method, when there is a choice between assigning window seat or aisle seat passengers of a row to an apron bus trip, the method assigns the window seat passenger to the earlier bus trip. For instance, in row 14, the window seat passengers take the first bus trip, and the aisle seat passengers take the (later) second bus trip. This adaptation will result in faster boarding times (and fewer seat interferences) than those resulting from the classical Back-to-front method that does not indicate a preference for window seat passengers boarding before aisle seat passengers.

**FIGURE 3.**
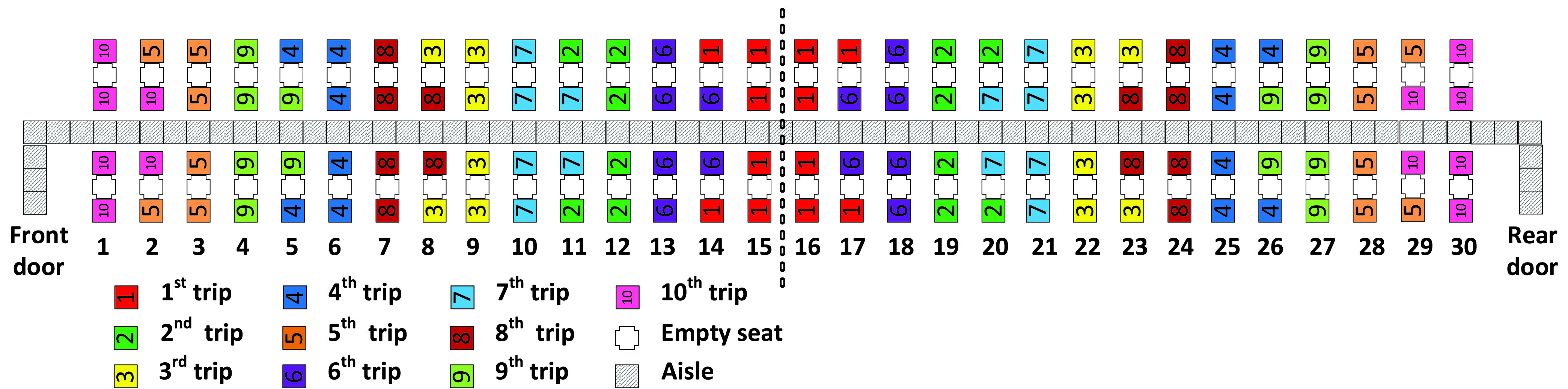
Adapted Back-to-front mix method.

Similarly, the Adapted Back-to-front mix method favors the assignment of window seat passengers to apron bus trips that board earlier than the trips that contain aisle seat passengers in the same row as illustrated in Figure 3. With the Adapted Back-to-front mix method, when passengers from the first five apron bus trips have taken their seats, there will be enough empty seats between each of those consecutively boarding first five trips to allow space for the passengers from the final five apron bus trips to sit. For instance, the passengers from the $6^{\mathrm {th}}$ apron bus trip will sit between passengers of the $1^{\mathrm {st}}$ and $2^{\mathrm {nd}}$ bus trips as illustrated in Figure 3.

### B. Adaptations of WilMA

The classical WilMA method boards passengers in three groups. First window seat passengers board, followed by middle seat passengers, and finally by aisle seat passengers. With the middle seat unoccupied due to social distancing, there are various possibilities for boarding consistently with the classical WilMA approach when there are ten boarding groups due to the ten apron bus trips.

We present three adaptations of the classical WilMA method. In each adaptation, all the window seat passengers travel to the airplane in the first five apron bus trips. They are followed by the final five bus trips that contain the aisle seat passengers.

**FIGURE 4.**
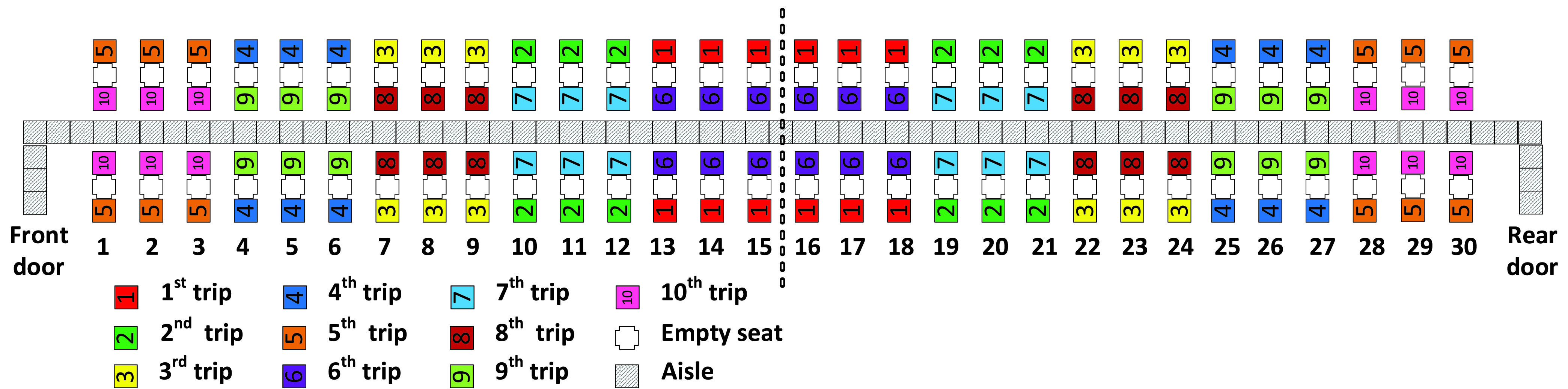
WilMA-Back-to-front method.

In the WilMA-Back-to-front method, the first apron bus trip contains those passengers with window seats closest to the middle of the airplane (in rows 13–18), followed a second bus trip containing passengers with window seats in rows 10–12 and 19–21, and so forth until all window seat passengers have boarded, as illustrated in Figure 4. The final five bus trips contain passengers with aisle seats in the same sequence. That is, the $6^{\mathrm {th}}$ apron bus trip has aisle seat passengers in rows 13–18, the $7^{\mathrm {th}}$ bus trip aisle seat passengers sitting in rows 10–12 and 19–21, and so forth. Thus, in the WilMA-Back-to-front method, the first priority is boarding window seat passengers before aisle seat passengers, and the second priority is to board passengers in a Back-to-front sequence.

**FIGURE 5.**
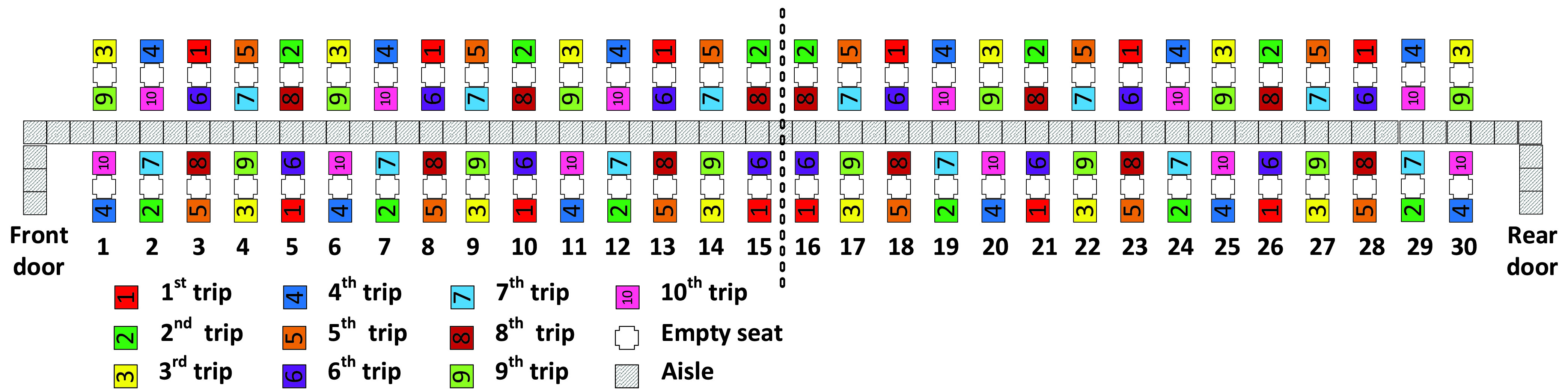
WilMA-Spread method.

The WilMA-spread method has a first priority is boarding window seat passengers first, and the second priority is spreading the passengers from each bus trip across different rows of the airplane. Observe in Figure 5, for instance, that the first apron bus trip carries one window seat passenger seated in each of the following rows: 3, 5, 8, 10, 13, 15, 16, 18, 21, 23, 26, and 28. Apron bus trips 2 through 5 likewise, carry passengers assigned to window seats that are spread throughout the airplane. Apron bus trips 6 through 10 each carry passengers assigned to aisle seats that are spread throughout the plane.

**FIGURE 6.**
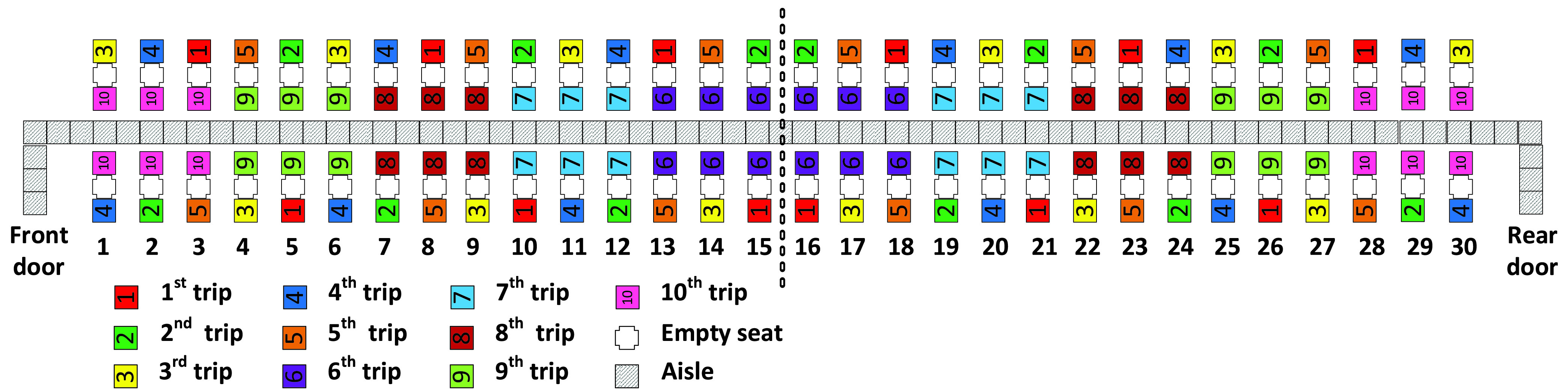
WilMA-Spread-Back-to-front method.

The WilMA-Spread-Back-to-front method behaves like WilMA-Spread for passengers with window seats, and like WilMA-Back-to-front for passengers with aisle seats. The corresponding boarding scheme is depicted in Figure 6.

### C. Adaptations of Reverse Pyramid

According to Nyquist and McFadden [43], van den Briel *et al.* [27] designed the Reverse pyramid method. In considering the van den Briel *et al.* [27] method for one-door airplanes, we observe that the first and last boarding groups of the Reverse pyramid method always contain only passengers in window seats (those closest to the rear of the airplane) and aisle seats (those closest to the front of the airplane) respectively, while the other boarding groups either have passengers with window and middle seats, or with middle and aisle seats. The pattern of those other boarding groups is sometimes called *diagonal*.

**FIGURE 7.**
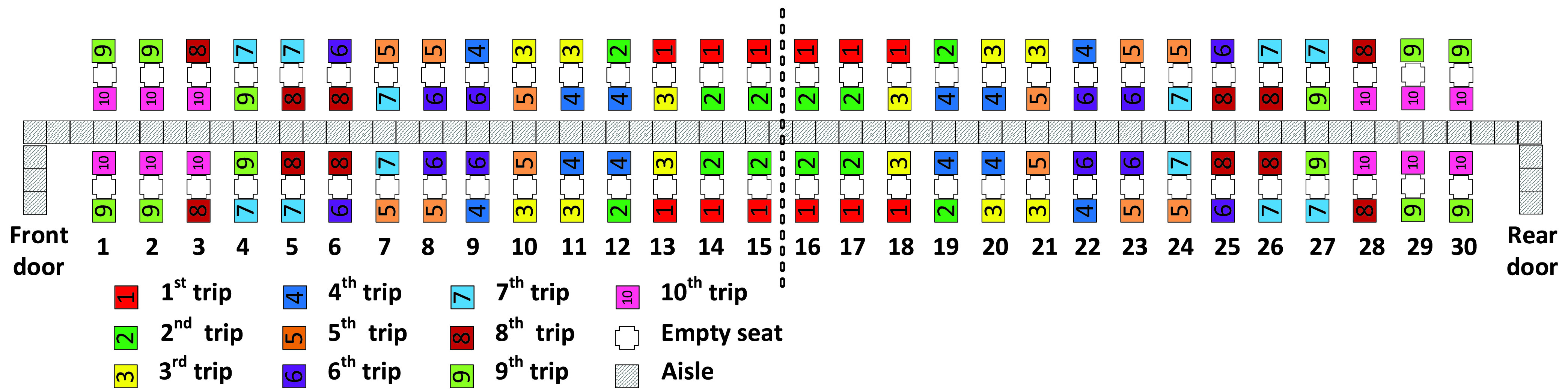
Adapted Reverse pyramid – Gradual method.

**FIGURE 8.**
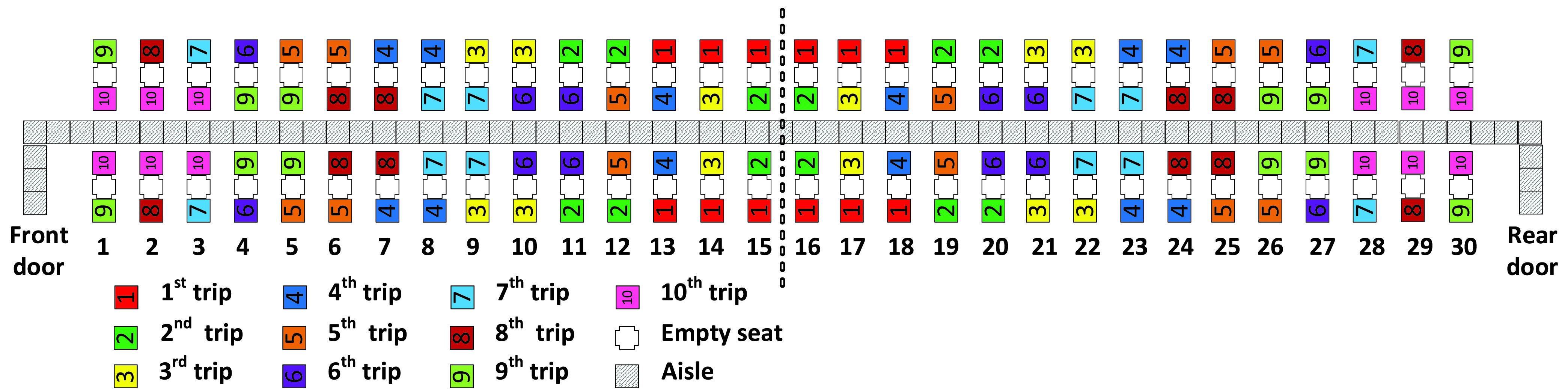
Adapted Reverse pyramid – Steep method.

**FIGURE 9.**
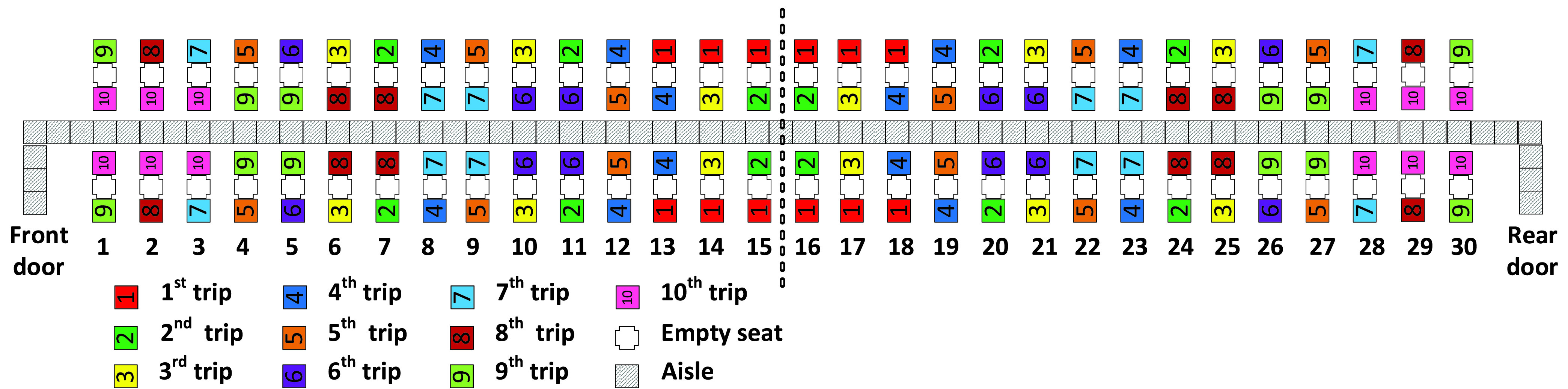
Adapted Reverse pyramid - Spread method.

In adapting the Reverse pyramid method for social distancing, we assign to the first apron bus trip the passengers with window seats in rows 13–18, and to the final (tenth) apron bus trip the passengers with aisle seats closest to a door (in rows 1–3 and rows 28–30). The prior work may imply that each of the other bus trips (2–9) should contain some passengers with window seats and other passengers in aisle seats—an approach that seems prudent to us—though it is less clear how many passengers of each seat position to assign to each bus. To investigate the latter, we propose (and later evaluate) three variations of adapted Reverse pyramid methods as we illustrate in Figures 7–9.

In the Reverse pyramid – Gradual boarding method of Figure 7, apron buses 2–9 alternate between carrying two aisle seat passengers and one window seat passenger and vice versa. This approach results in a “gradual” allocation of window seat passengers to the apron bus trips as the number of assigned trips increases to five. This contrasts with the “steeper” (more rapid) allocation of window seat passengers to the first five apron bus trips that is illustrated in Figure 8 (We might characterize the latter method as having a “steeper diagonal”).

With the Reverse pyramid – Steep method, the first apron bus trip carries passengers who have window seats closest to the middle of the airplane (rows 13–18). The next four apron bus trips each carry eight passengers with window seats (and four passengers with aisle seats) that are as close to the middle of the airplane as possible. The following four apron bus trips each carry eight passengers with aisle seats (and four passengers with window seats) that are as close to the middle of the airplane as possible. The final bus trip (the $10^{\mathrm {th}}$ trip) contains the 12 passengers with the aisle seats that are closest to an airplane door.

Figure 9 illustrates the Reverse pyramid – Spread method. This method assigns the aisle seat passengers to the same apron bus trips as the Reverse pyramid – Steep method, but tends to assign to apron bus trips passengers sitting in window seats that are more spread out across the rows than in the other method. For instance, in the Reverse pyramid – Spread method, the fifth bus trip contains passengers who have seats in rows 4, 9, 12, 19, 22, and 27. The motivation for the spread variation is to increase the probability that some pairs of passengers traveling on the same apron bus may take their seats simultaneously and thus reduce the boarding time.

All three adapted Reverse pyramid methods have the property that within each row, the window seat passengers travel in an earlier bus trip than the aisle seat passengers. This same property is shared by the WilMA method and its adaptations. Furthermore, with the adapted Reverse pyramid methods, both aisle seat passengers of every row travel in the same apron bus trip. We experimented with some variations that did not follow the latter property and found that they led to increased health risk of seated aisle seat passengers being passed by later boarding passengers walking near them; that increased health risk was not offset by a commensurably favorable increase in the other performance metrics. In this article, we do not present those adapted Reverse pyramid methods that perform worse than the three we include.

For all three adapted Reverse pyramid methods, the aisle seat passengers board in a back-to-front sequence. Meanwhile, in the Reverse pyramid – Gradual method and the Reverse pyramid – Steep method, the window seat passengers board in a back-to-front sequence, but this is not the case for the Reverse pyramid – Spread method. In summary, the adapted Reverse pyramid methods contain favorable aspects of WilMA, Reverse pyramid, Back-to-front, and for the one adaptation, spreading.

## IV. Metrics and Scenarios

To evaluate the boarding methods and to compare their effectiveness in terms of health risk and boarding time performance, four metrics will be used [18] and seven scenarios considered with respect to the frequencies of luggage carried by the passengers boarding into the airplane [3], [18].

### A. Metrics

The first metric refers to the time needed to complete the boarding, expressed in seconds. This boarding time represents the amount of time between the moment when the first passenger enters the airplane, using either the front or rear door, and the moment the last passenger takes has sat down.

**FIGURE 10.**
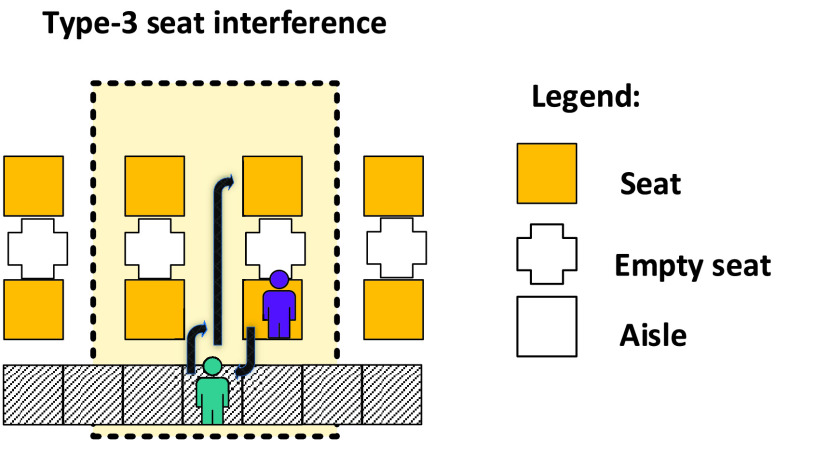
Type-3 seat interference results from a passenger (purple) in an aisle seat needing to stand to enable a later boarding passenger (bluish green) to sit in the window seat.

The other three metrics refer to the health and safety of the passengers during the boarding process as described in [18]. The total number of seat interferences counts the situations in which the type-3 seat interference occurs. A seat interference arises when a passenger having a window (or middle) seat needs to rise to clear the path for a later boarding window (or aisle) seat passenger to sit. In general, four types of seat interferences can be encountered [51]. However, in the special situation generated by the COVID-19 restrictions which require the middle seat to be unoccupied, only type-3 seat interference can occur. As depicted in Figure 10, the type-3 seat interference appears when a passenger already seated on an aisle seat needs to clear the path for a passenger having a window seat in the same row. When this happens, it is possible for either passenger—if contagious—to shed the virus to the other passenger or to nearby seated or standing passengers. The transmission could happen directly through the air or by the mutual touching of an armrest or headrest.

Another health metric refers to the risk generated by possibly contagious passengers moving down the aisle to their assigned seats while passing the row in which passengers are already seated in an aisle seat (aisle seat risk) or window seat (window seat risk). The greater the total duration of these exposures, the higher the health risk. These two metrics are measured in seconds based on the following formulas [18]:

\begin{align*}&\hspace {-2pc}AisleSeatRisk \\=&\sum \limits _{p} \sum \limits _{r\le RowSit_{p}} \left ({ RowTime_{pr}\ast \sum \limits _{p^{\prime }< p} {AisleSeat_{p^{\prime }r}} }\right )\\&\hspace {-2pc}WindowSeatRisk \\=&\sum \limits _{p} \sum \limits _{r\le RowSit_{p}} \left ({ RowTime_{pr}\ast \sum \limits _{p^{\prime }< p} {WindowSeat_{p^{\prime }r}} }\right )\end{align*}

where:

$p =$ passenger advancing towards his/her seat
$r =$ row index
*RowSit*$_{p} =$ row in which passenger $p$ has a seat
*RowTime*$_{pr} =$ time that passenger $p$ spends in row $r$ (this duration begins when passenger $p$ begins to enter row $r$ and concludes when passenger $p$ begins to leave row $r$; this convention is chosen because a passenger’s nose and mouth are at the front of the passenger)
  \begin{align*} p'=&passenger boarding before passenger p\\&\hspace {-2pc}{AisleSeat}_{p'r} \\=&\begin{cases} 1& \text {if passenger' has an aisle seat in row } r \\ 0& \text {otherwise} \\ \end{cases}\\&\hspace {-2pc}{WindowSeat}_{p'r} \\=&\begin{cases} 1& \text {if passenger } p' \text {has an window seat in row }r \\ 0& \text {otherwise} \\ \end{cases}\end{align*}

### B. Luggage Scenarios

Seven luggage situations are considered as suggested by [1], [6], [42]. The luggage situations feature different frequencies of the bags carried by the passengers for no luggage, one small, two small, one large, one large and one small luggage cases. The frequencies (percentages) are presented in TABLE 2. While the number of passengers carrying each combination of bags is determined from the luggage situation, the particular passengers with each amount of carry-on luggage is determined randomly.

**TABLE 2:** The Considered Luggage Situations

Situation	Percentages of bags carried by the passengers				
	0 bag	1 small bag	2 small bags	1 large bag	1 large and 1 small bag
S1	10%	10%	0%	10%	70%
S2	15%	20%	5%	10%	50%
S3	25%	20%	10%	15%	30%
S4	35%	25%	10%	15%	15%
S5	60%	10%	10%	10%	10%
S6	80%	5%	5%	5%	5%
S7	100%	0%	0%	0%	0%

## V. Agent-Based Modeling of the Methods

Agent-based modeling has been frequently used in the literature for reflecting passengers’ behavior while boarding an airplane [2], [3], [5]. In a recent paper, Currie *et al.* [52] assert that agent based modelling is appropriate for modeling human behavior. The authors assert that variability is easy to incorporate in such models, while simulation of small changes in the initial values of the parameters can offer hints regarding the corresponding levels of output. These observations are consistent with other works from the field [53]–[58]. Among the agent-based platforms, NetLogo [59] has attracted the attention of researchers from different areas [60]–[62]. It provides an intuitive and easy to write programming code section, a visual interface, integrated graphics, real-time user access on an agent’s state, and it is free to use [18].

### A. Agents Characteristics

To model the process of passengers boarding an airplane using two doors in the presence of apron buses and accounting for social distancing, we use two types of agents.

The first type of agents is represented by the “patches” which are small rectangular pieces that form the NetLogo “world”.

**FIGURE 11.**
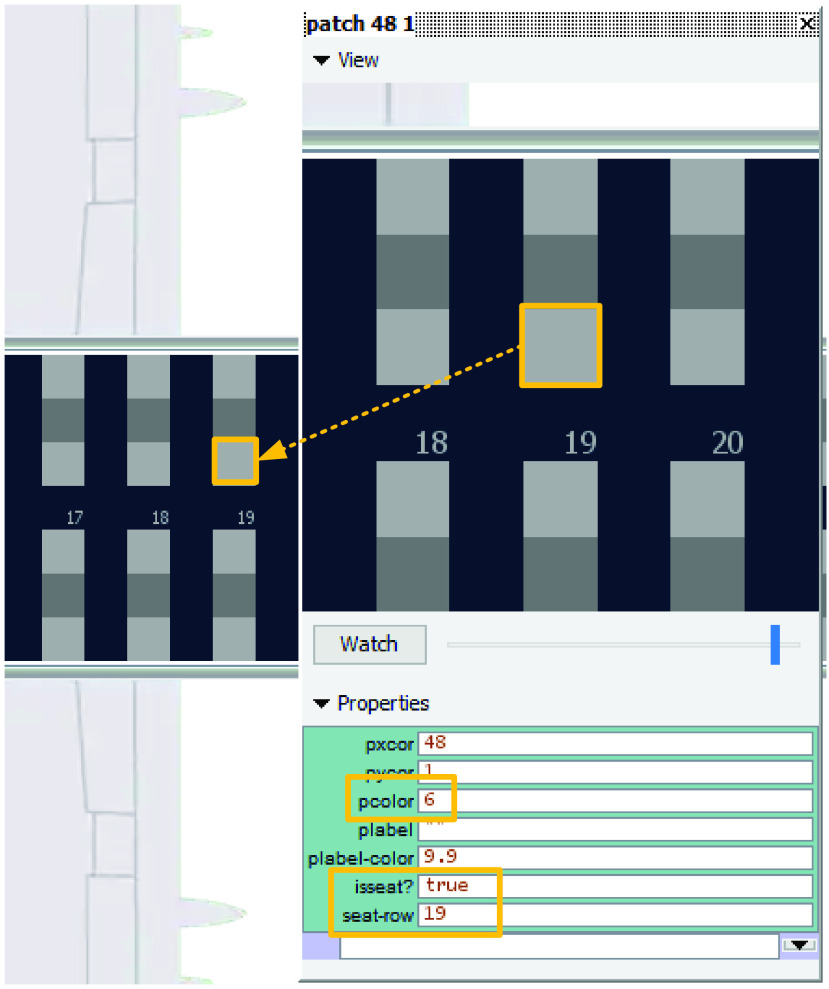
Patches properties (example for patch 48 1).

The patches represent the aisles and seats of the airplane and possess different characteristics as highlighted in Figure 11: *pcolor*, *isseat?* and *seat-row*. *Pcolor* represents the color of the patch which can be either grey (in two tones of grey) for seats or dark blue for aisles. *Isseat?* can take either a true value (indicating the patch represents a seat) or a false value (indicating the patch represents a portion of the aisle). *Seat-row* takes values between 1 and the number of rows of the airplane and indicates the row in which the passenger has a seat. The dimension of a patch is equivalent to 0.4 meters $\times$ 0.4 meters as suggested by [63], [64].

**FIGURE 12.**
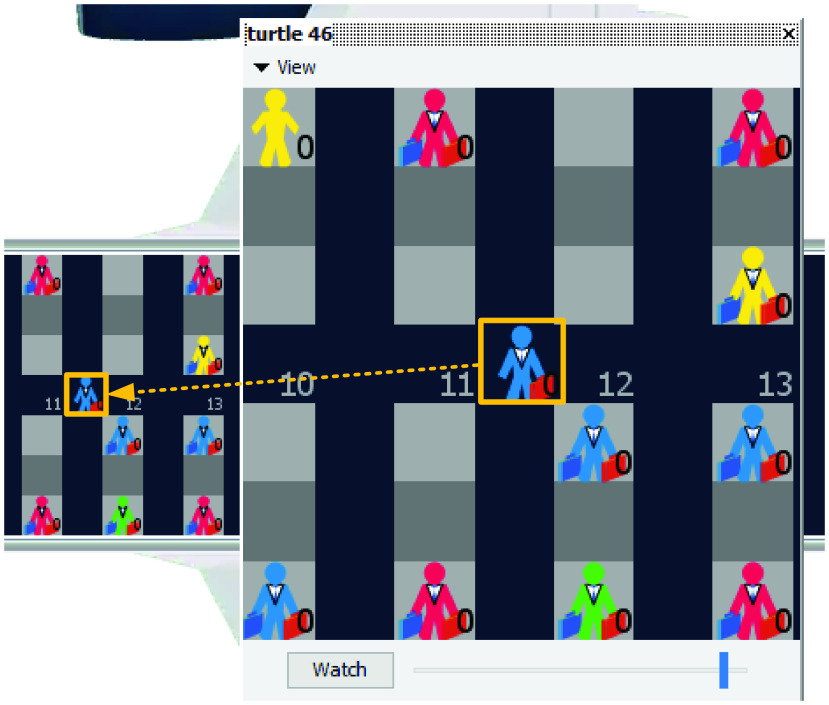
Close view of turtle agent number 46.

Another type of agents is represented by the “turtles” that represent the passengers heading to their assigned seats and that possess a series of characteristics so that these agents model the passengers involved in the boarding process. Figure 12 presents a close view of a randomly selected turtle agent that carries a large hand luggage (the small hand luggage is represented using blue color, while the large hand luggage is represented by red color).

A series of properties have been assigned to the turtle agents [1], [3], [5]: *speed*, *luggage?*, *large-luggage*, *small-luggage*, *luggage-store-time*, *bus*, *seated?*, *agent-seat-row*, *agent-seat-column*, *aisle*-*social-distance*, *time-to-seat*.

*Speed* ranges between 0 and 1 patches/tick (tick is the time unit in NetLogo), which is equivalent to a speed of up to 0.33 m/s [42], [64], [65]. The maximum speed is reached when the agent has no luggage and its flow is not impeded by another agent in front of it. An agent progressing down the aisle will slow or stop, if necessary, to maintain the minimum *aisle-social-distance* between it and any agent in front of it. When an agent carries luggage, its speed is determined using the uniform probability distribution with a range between 0.6 patches/tick and 0.9 patches/tick [1], [3], [5].

**FIGURE 13.**
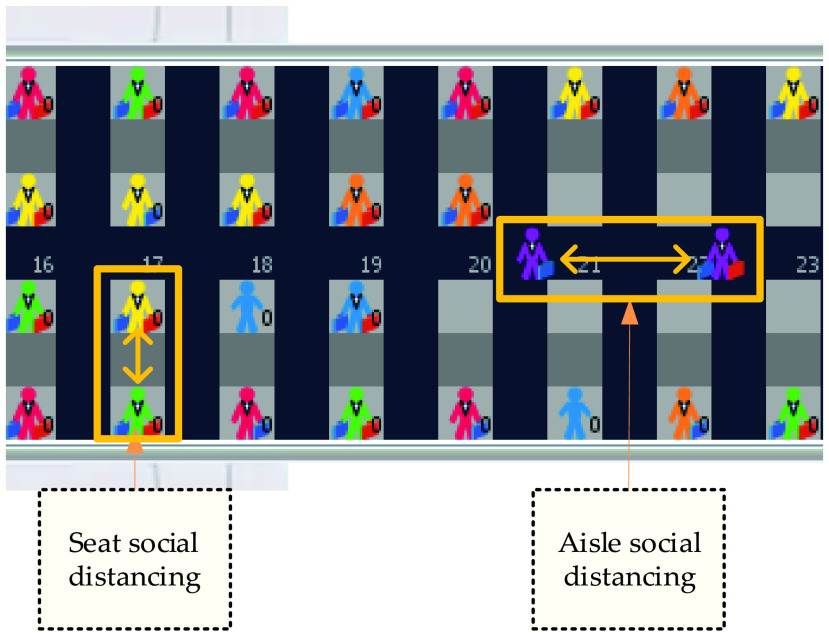
Preserving seat and aisle social distancing as indicated by the double-headed orange arrows.

*Luggage?* is a true/false variable that indicates whether the agent is travelling with or without luggage. Two other variables, *large-luggage* and *small-luggage*, refer to the number of small and large luggage the agent brings inside the airplane. The *large-luggage* variable can take 0 or 1 as values, while *small-luggage* can be either 0, 1 or 2. The *luggage-store-time* variable represents the time to store luggage as calculated using the formula suggested by [66] and used in [1], [3], [6], [22], [42]:

\begin{align*} Tstore=&( (NbinLarge+0.5 NbinSmall+NpassengerLarge \\&+\,0.5NpassengerSmall )\ast (NpassengerLarge \\&+\,0.5 NPassengerSmall)/2)\ast Trow\end{align*}

where:

*Tstore* is the time to store the luggage
*NbinLarge* is the number of large bags in the bin prior to the passenger’s arrival
*NbinSmall* is the number of small bags in the bin prior to the passenger’s arrival
*NpassengerLarge* is the number of large bags carried by the passenger
*NpassengerSmall* is the number of small bags carried by the passenger
*Trow* is the time for a passenger to walk from one row to the next (when not delayed by another passenger in front)
*Bus* represents the apron bus trip to which an agent is assigned and it has a value between 1 and 10.
*Seated?* is a true/false variable. When true, it symbolizes that the agent has occupied its assigned seat.
*Agent-seat-row* and *agent-seat-column* indicates the seat row (numbered from 1 to 30) and the column (marked with A, C, D or F letters) in which the seat of the agent is allocated. With the COVID-19 imposed social distancing norms, the columns marked with B and E letters are not available to preserve seat social distancing as presented in Figure 13.
*Aisle-social-distance* is a variable introduced in the context of the social distancing imposed by the COVID-19 outbreak and represents the distance between the agents while advancing in the aisle to their seats (please see Figure 13). The values for the *aisle-social-distance* are reported in meters and can be: 1 m or 2 m [18].
*Time-to-seat* is equal to 1 tick and it represents the time needed by an agent standing in its seat’s row to take its seat in the absence of seat interferences.

**FIGURE 14.**
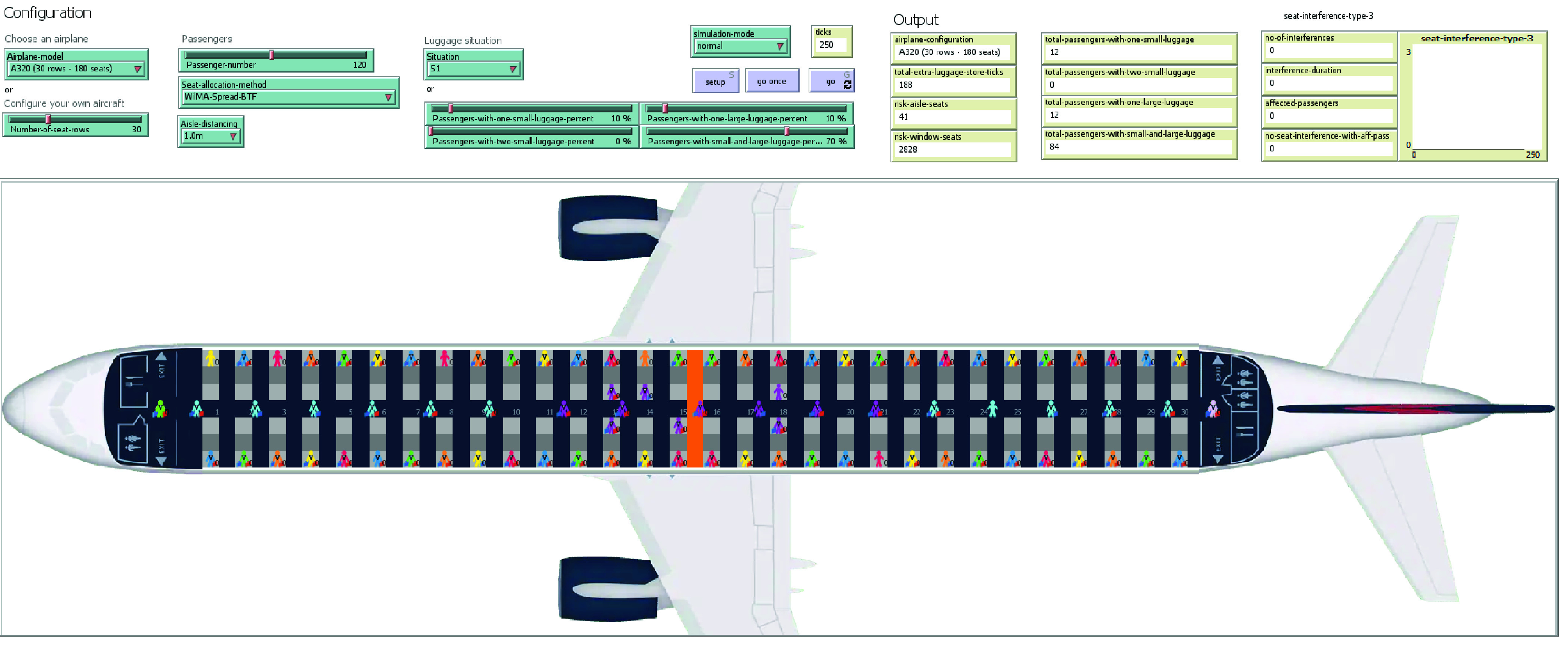
The GUI for the agent-based model in NetLogo 6.1.1 (example of view for WilMA-Spread-Back-to-front method with 1 m social distancing).

The model built in NetLogo 6.1.1 is configurable, and the graphical user interface (GUI) is presented in Figure 14.

To compare the relative performance of the proposed boarding methods, the values of four metrices are displayed in the output area under the headings: *ticks*, *no-of-interferences*, *risk-aisle-seats* and *risk-window-seats*.

### B. Other Assumptions on Rules of Movement

For the boarding process, we make a series of assumptions as presented in the following. First, the number of apron bus trips needed for the passengers’ transport between the airport terminal and the airplane is equal to 10, each of them accommodating 12 passengers. The 10 trips could be conducted using any number of apron buses (e.g. one apron bus making ten trips, ten apron buses making one trip each). The assignment of the passengers to the buses is made according to the boarding methods previously explained in Section 3.

We assume the flow of passengers is continual, meaning that once the last passengers from the first apron bus trip have entered the airplane, other passengers from the second bus trip have arrived and are waiting to enter the aisle through the front and rear doors, maintaining the imposed social distance. Passengers enter the cabin through the airplane’s front door simultaneously with other passengers entering the cabin through the rear door.

We assume that the airlines provide clear descriptions to the passengers regarding the bus they should take and the airplane door they should enter, for example, by using panels in which the name or the passengers’ seat is displayed, or recording on the boarding pass the required information. Similarly, we assume that every passenger takes the assigned bus and enters the airplane through the assigned door. The 12 passengers exiting an apron bus board the airplane in a random sequence. Of course, as with any procedure involving human beings, instructions are not always followed and when this happens, performance worsens. Unpredictable human misbehavior affects all of the examined boarding methods and conditions, so our assumptions do not favor one method versus another.

We assume the time for a seat interference is triangularly distributed with a mode of 10 seconds and minimum and maximum values of 9 seconds and 13 seconds respectively [3], [64].

## VI. Simulations and Results

We used the BehaviourSpace tool provided by NetLogo [56] for simulating the considered situations for 1 m and 2 m aisle social distancing. For each experimental condition, 10,000 simulation trials have been run and the rounded average results are presented and analyzed in the following for each of the four-performance metrics.

### A. Results for Boarding Time

The average times to complete boarding for the nine methods and seven luggage situations, while considering 1 m aisle social distancing, are shown in TABLE 3. The best performing boarding time for each luggage situation is in **bold** font to facilitate interpretation. With 2 m aisle social distancing, the average boarding times are shown in TABLE 4.

**TABLE 3:** Average Boarding Time With Blocked Middle Seats and 1 m Aisle Social Distancing (in Seconds)

Boarding method	Aisle social distancing: 1 m						
	S1	S2	S3	S4	S5	S6	S7
Random	582	555	522	491	455	414	357
Adapted Back-to-front	652	600	548	502	459	410	348
Adapted Back-to-front mix	616	566	522	480	439	396	328
WilMA-Back-to-front	531	488	447	412	377	333	254
WilMA-Spread	488	461	435	407	374	333	272
WilMA-Spread-Back-to-front	508	470	434	402	365	323	254
Reverse pyramid - Gradual	510	474	438	407	374	332	254
Reverse pyramid - Steep	488	455	426	397	367	326	254
Reverse pyramid - Spread	476	448	420	392	364	323	254

**TABLE 4:** Average Boarding Time With Blocked Middle Seats and 2 m Aisle Social Distancing (in Seconds)

Boarding method	Aisle social distancing: 2 m						
	S1	S2	S3	S4	S5	S6	S7
Random	835	793	751	712	657	601	530
Adapted Back-to-front	897	840	777	726	669	601	519
Adapted Back-to-front mix	853	803	749	702	645	580	498
WilMA-Back-to-front	785	731	680	633	578	511	414
WilMA-Spread	726	691	653	618	565	507	429
WilMA-Spread-Back-to-front	759	712	663	618	564	500	414
Reverse pyramid – Gradual	770	720	672	628	573	508	414
Reverse pyramid – Steep	734	693	654	615	565	503	414
Reverse pyramid - Spread	729	689	650	612	562	501	414

Unsurprisingly, for all methods, the average boarding times are higher when more luggage is carried aboard the airplane. It takes time to store luggage, and more luggage leads to longer lasting queues of passengers waiting in the aisle.

For all luggage and aisle social distancing situations, the methods with the longest boarding times are Random, Adapted Back-to-front, and Adapted Back-to-front mix. When no luggage is carried aboard the airplane (luggage scenario S7), five methods perform equally well.

Of the WilMA methods, WilMA-Spread has the best average boarding times for high volumes of luggage—S1 and S2 with 1 m aisle social distancing, and S1, S2, S3, and tied for the best WilMA for S4 with 2 m aisle social distancing—and WilMA-Spread-Back-to-front has the best WilMA boarding times when lower volumes of luggage are carried aboard the airplane.

**FIGURE 15.**
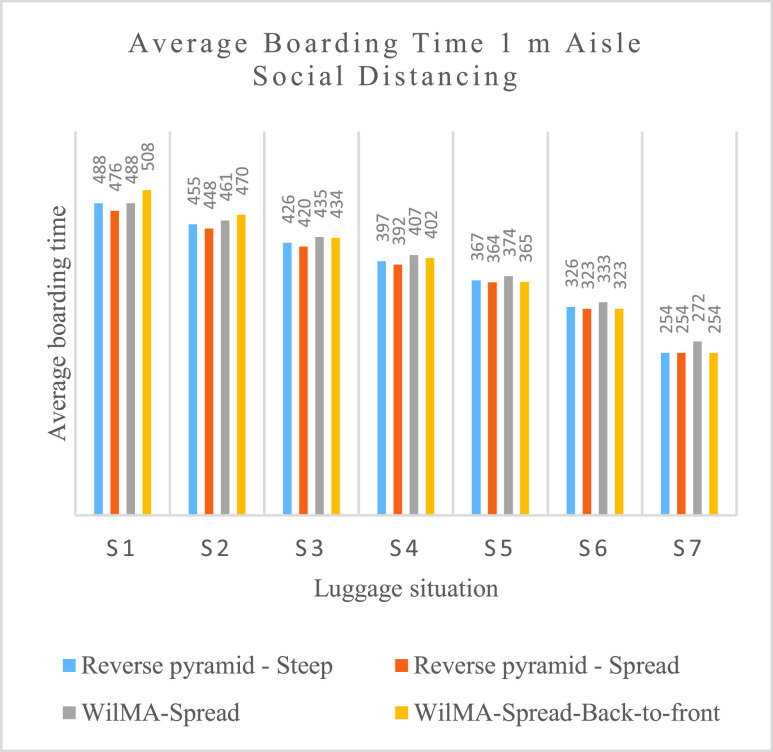
Average boarding time for 1 m social distancing for four methods (in seconds).

Of all nine boarding methods, Reverse pyramid – Spread has the best average boarding times with 1 m aisle social distancing, followed closely by Reverse-pyramid – Steep and the best of the WilMA methods as illustrated in Figure 15.

The Reverse pyramid – Spread results in the greatest reduction in boarding time of 12 seconds (which is a 2.52% reduction) from the boarding time of its two closest competing methods for the S1 luggage situation. Its advantage decreases for the lower volume luggage situations S2 through S5 and is eliminated entirely for the lowest luggage situations S6 and S7.

Because of the inherent statistical fluctuations of stochastic simulations, small differences in performance may stem more from randomness than anything else. Still, an assignment of passengers spread across multiple rows to a same apron bus trip facilitates the possibility of pair(s) of them storing their luggage at the same time. When simultaneous luggage storage occurs, this should reduce boarding time. Consequently, it is not surprising that when volumes of luggage are high, Reverse pyramid – Spread has the best average boarding times, and that of the adapted WilMA methods, WilMA – Spread has the best average boarding times followed by WilMA – Spread-Back-to-front. We conclude that spreading helps slightly with boarding time when passengers carry a lot of luggage aboard the airplane.

If no passengers have luggage (the S7 scenario), there is no advantage from spreading. In fact, with no passenger luggage, the WilMA – Spread method has the longest average boarding time of the WilMA methods—probably because of its disadvantage of having passengers from the $10^{\mathrm {th}}$ apron bus trip spread across more rows of the airplane resulting in them being further from the nearest airplane door.

With 2 m aisle social distancing, of the nine boarding methods, the Reverse pyramid – Spread method and the best of the WilMA methods have the best (and essentially the same) average boarding times across all luggage situations—with times slightly better than those resulting from the Reverse pyramid – Steep method for all but the no luggage scenario (S7). With the passengers proceeding towards their seats being separated by 2 m aisle social distancing, the probabilities of simultaneous luggage storage is reduced with the spread-based methods, and thus their relative advantage in high volume luggage situations is reduced compared with their relative performance with 1 m aisle social distance.

Unsurprisingly, the average boarding times of all methods and all luggage situations increases considerably when the aisle social distance is increased from 1 m to 2 m.

### B. Results for Seat Interferences

As discussed above in section IV and depicted in Figure 10, the average number of seat interferences is a key health metric due to their potential for virus transmission. The number of seat interferences for the 7 luggage situations and with 1 m and 2 m aisle social distancing are shown in TABLE 5 and TABLE 6. For each of the boarding methods, the number of seating interferences does not depend on either the luggage carried or the magnitude of the aisle social distance.

**TABLE 5:** Average Total Number of Seat Interferences With Blocked Middle Seats and 1 m Aisle Social Distancing

Boarding method	Aisle social distancing: 1 m						
	S1	S2	S3	S4	S5	S6	S7
Random	30	30	30	30	30	30	30
Adapted Back-to-front	20	20	20	20	20	20	20
Adapted Back-to-front mix	20	20	20	20	20	20	20
WilMA-Back-to-front	0	0	0	0	0	0	0
WilMA-Spread	0	0	0	0	0	0	0
WilMA-Spread-Back-to-front	0	0	0	0	0	0	0
Reverse pyramid - Gradual	0	0	0	0	0	0	0
Reverse pyramid - Steep	0	0	0	0	0	0	0
Reverse pyramid - Spread	0	0	0	0	0	0	0

**TABLE 6:** Average Total Number of Seat Interferences With Blocked Middle Seats and 2 m Aisle Social Distancing

Boarding method	Aisle social distancing: 2 m						
	S1	S2	S3	S4	S5	S6	S7
Random	30	30	30	30	30	30	30
Adapted Back-to-front	20	20	20	20	20	20	20
Adapted Back-to-front mix	20	20	20	20	20	20	20
WilMA-Back-to-front	0	0	0	0	0	0	0
WilMA-Spread	0	0	0	0	0	0	0
WilMA-Spread-Back-to-front	0	0	0	0	0	0	0
Reverse pyramid - Gradual	0	0	0	0	0	0	0
Reverse pyramid - Steep	0	0	0	0	0	0	0
Reverse pyramid - Spread	0	0	0	0	0	0	0

With the Random boarding method, for a given row and side of the airplane, there is a 50% probability that the aisle seat passenger be seated prior the window seat passenger resulting in a seat interference. With 30 rows and two sides of the airplane, we would expect there to be 30 seat interferences with the Random boarding method as we see in in TABLE 5 and TABLE 6.

For the Adapted Back-to-front and Adapted-Back-to-front-mix boarding methods illustrated in Figure 2 and in Figure 3, recall that when there was a choice between assigning an aisle seat passenger or a window seat passenger to a particular apron bus trip, we chose to always assign the window seat passenger to the earlier bus trip. For both methods, this occurred for 10 of the 30 airplane rows. Consequently, we would expect the Adapted Back-to-front and Adapted-Back-to-front-mix boarding methods to have average a total of 20 seat interferences as we see in TABLE 5 and TABLE 6.

For the three adapted WilMA methods and the three adapted Reverse pyramid methods, in a particular row and side of the airplane, the window seat passenger always boards an earlier apron bus trip than the aisle seat passenger thus leading to no seat interferences for these six methods. Consequently, these six methods outperform the others from the perspective of the health metric of average number of seat interferences.

### C. Results for Aisle Seat Risk

As potentially contagious passengers walk down the aisle toward their assigned seats (or queueing in the aisle), they may spread coronavirus to passengers already seated in rows they traverse. Because aisle seat passengers border the aisle, they are close to passengers walking by them. As described above in section IV, the *aisle seat risk* measures the total duration that aisle seat passengers have a later boarding passenger walking or standing near them in the aisle in the seated passenger’s row. The smaller the value of aisle seat risk, the lower the risk of infecting aisle seat passengers.

The aisle seat risks obtained for 1 m and 2 m aisle social distance are shown in TABLE 7 and TABLE 8. For all methods, the aisle seat risk durations are higher when more luggage is carried onto the airplane (e.g. luggage situation S1).

**TABLE 7:** Average Aisle Seat Risk Duration With Blocked Middle Seats and 1 m Aisle Social Distancing (in Seconds)

Boarding method	Aisle social distancing: 1 m						
	S1	S2	S3	S4	S5	S6	S7
Random	4699	4522	4223	3972	3696	3413	3076
Adapted Back-to-front	858	781	691	620	565	522	500
Adapted Back-to-front mix	2143	1952	1786	1618	1466	1336	1190
WilMA-Back-to-front	455	397	348	302	260	229	199
WilMA-Spread	1592	1498	1403	1313	1192	1074	941
WilMA-Spread-Back-to-front	455	401	349	302	263	229	198
Reverse pyramid - Gradual	316	271	232	196	166	141	121
Reverse pyramid - Steep	317	271	230	191	166	142	122
Reverse pyramid - Spread	316	273	230	196	165	142	121

**TABLE 8:** Average Aisle Seat Risk Duration With Blocked Middle Seats and 2 m Aisle Social Distancing (in Seconds)

Boarding method	Aisle social distancing: 2 m						
	S1	S2	S3	S4	S5	S6	S7
Random	4464	4256	3966	3762	3496	3223	2969
Adapted Back-to-front	836	765	678	618	573	526	500
Adapted Back-to-front mix	1652	1564	1472	1387	1276	1179	1078
WilMA-Back-to-front	402	360	318	284	250	220	198
WilMA-Spread	1546	1464	1364	1286	1174	1056	938
WilMA-Spread-Back-to-front	401	358	321	285	251	221	199
Reverse pyramid - Gradual	298	259	221	188	163	139	121
Reverse pyramid - Steep	301	258	221	188	162	142	122
Reverse pyramid - Spread	300	258	221	186	163	140	121

**FIGURE 16.**
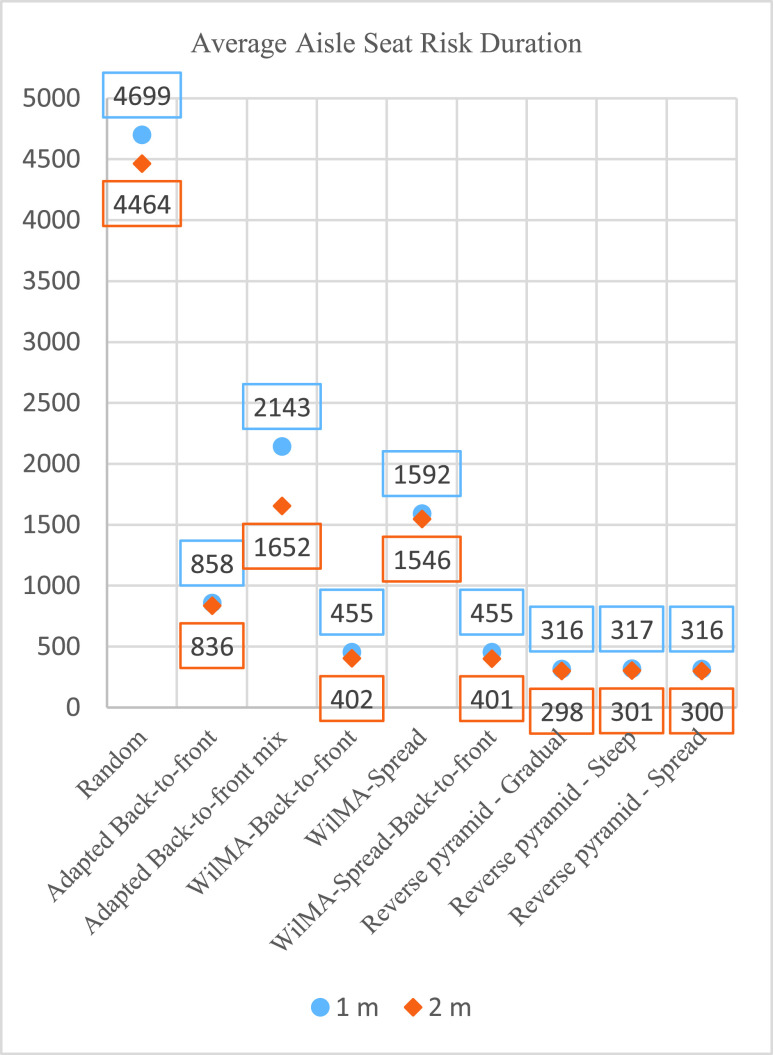
Average aisle seat risk durations for 1 m and 2 m aisle social distancing (in seconds).

For all luggage-carrying situations, the resulting aisle seat risk is lower with 2 m aisle social distancing than with 1 m aisle social distancing (with the differences larger for the methods with the worst aisle seat risk durations as illustrated in Figure 16). This makes sense. When a walking passenger is 2 m behind another passenger in the aisle, once the latter passenger has departed the aisle, the former passenger has at least two unobstructed meters of open aisle to traverse before potentially encountering another delay. This enables the walking passenger to proceed without any delay for those two meters and thus walk by any previously seated passengers in those rows without delay. Conversely, with 1 m aisle social distancing, the walking passenger can be assured only of one meter of unobstructed walking.

With both 1 m and 2 m aisle social distances, the best performing methods are the three adaptations of the Reverse pyramid methods. All three Reverse pyramid methods have aisle seat risk durations that are close to each other, with there being no discernible pattern of difference among them other than that caused by the inherent variability of stochastic simulation.

For each luggage situation with 1 m aisle social distancing, the best Reverse pyramid method has between 44% and 64% less aisle seat risk than the best of the methods that are not adapted from Reverse pyramid. With 2 m aisle social distancing, the reduction drops to between 26% and 39%.

The Reverse Pyramid methods—as adapted and not arbitrarily in this regard—have low aisle seat risks for several reasons. Given that the Reverse pyramid methods assign only window (and aisle) seat passengers to the first (and last) apron bus trips, the three adapted methods assign the remaining passengers to the apron bus trips to maintain several properties. For apron bus trips 2–9, each has either four or eight aisle seat passengers. For every row, both aisle seat passengers take the same apron bus trip. The aisle seat passengers are assigned to bus trips 2–9 in a back-to-front sequence. If this were not the case, aisle seat risk would be higher. If any of bus trips 2–9 had all its passengers seated in aisle seats, then the total number of occurrences of aisle seat risk—an occurrence being defined as when a passenger walks into the row of a previously seated passenger—would be higher. To understand why, please consider two aisle seat passengers assigned to a same row. Exactly one of them will board the airplane first resulting in one occurrence of aisle seat risk, that is, one occurrence of aisle seat risk per row. Now consider two consecutive rows of aisle seat passengers assigned to one apron bus trip. On average, for the row closest to an airplane door, its two passengers will each have half of one occurrence of aisle seat risk from each other and will also encounter half of one occurrence of aisle seat risk from each of the two passengers from the row further from the airplane door. In total, the average number of occurrences of aisle seat risk for that row closest to the door will be three and for the other row one, which is two occurrences per row for those two consecutive rows. The number of aisle seat risk occurrences per row worsens in the event of three consecutive rows of passengers having aisle seat passengers assigned to the same apron bus. The foregoing provides insight into why the assignment to bus trips 2–9 of exactly four or eight aisle seat passengers results in low aisle seat risk.

### D. Results for Window Seat Risk

Because window seat passengers are further from the aisle than aisle seat passengers, their risk of infection from later boarding passengers is considerably less. Consequently, window seat risk is less important than aisle seat risk. We leave it to infectious disease experts to determine how much less.

As shown in TABLE 9 and TABLE 10, the Adapted Back-to-front method has considerably lower average window seat risk than all the other eight boarding methods in all luggage situations and with 1 m and 2 m aisle social distancing. The second-best performing method for this metric is Reverse pyramid – Gradual which has between 70% and 86% higher window seat risk than Adapted Back-to-front for each luggage situation and with 1 m and 2 m aisle social distancing.

**TABLE 9:** Average Window Seat Risk Duration With Blocked Middle Seats and 1 m Aisle Social Distancing (in Seconds)

Boarding method	Aisle social distancing: 1 m						
	S1	S2	S3	S4	S5	S6	S7
Random	4414	4201	3905	3665	3364	3121	2790
Adapted Back-to-front	1091	947	821	708	627	555	505
Adapted Back-to-front mix	3752	3398	3078	2769	2484	2219	1920
WilMA-Back-to-front	5487	4886	4348	3891	3438	2977	2431
WilMA-Spread	5631	5272	4918	4584	4162	3729	3229
WilMA-Spread-Back-to-front	6738	6089	5512	5023	4486	3936	3230
Reverse pyramid - Gradual	1989	1738	1510	1313	1134	985	858
Reverse pyramid - Steep	2966	2663	2370	2106	1869	1643	1430
Reverse pyramid - Spread	3361	3023	2705	2435	2165	1900	1646

**TABLE 10:** Average Window Seat Risk Duration With Blocked Middle Seats and 2 m Aisle Social Distancing (in Seconds)

Boarding method	Aisle social distancing: 2 m						
	S1	S2	S3	S4	S5	S6	S7
Random	4177	3964	3716	3493	3203	2925	2666
Adapted Back-to-front	1041	908	785	677	602	526	468
Adapted Back-to-front mix	2931	2706	2480	2293	2069	1857	1625
WilMA-Back-to-front	4940	4470	4045	3659	3257	2859	2430
WilMA-Spread	5449	5120	4777	4477	4051	3653	3231
WilMA-Spread-Back-to-front	6126	5630	5162	4729	4250	3758	3228
Reverse pyramid - Gradual	1859	1644	1445	1260	1104	971	859
Reverse pyramid - Steep	2771	2502	2259	2034	1818	1615	1430
Reverse pyramid - Spread	3124	2852	2577	2334	2088	1863	1643

The third and fourth best performing methods for this metric are Reverse pyramid – Steep and Reverse pyramid – Spread.

**FIGURE 17.**
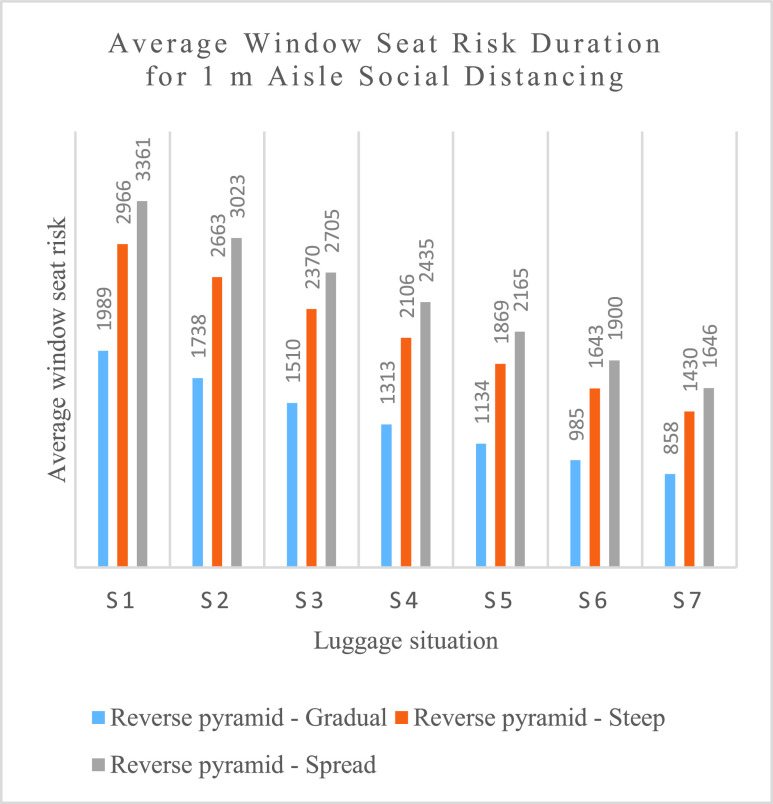
Average window seat risk duration for 1 m aisle social distancing (in seconds).

The average window seat risk durations of the Reverse pyramid methods with 1 m aisle social distancing are illustrated in Figure 17.

As with aisle seat risk, when the aisle social distancing is increased from 1 m to 2 m, the window seat risks decrease for all methods and all situations when luggage is carried aboard the airplane. For the S7 no luggage scenario, there is no meaningful difference in window seat risk for the six boarding methods based on WilMA and Reverse pyramid.

## VII. Concluding Remarks

In this article, we consider social distancing that reduces the capacity of apron buses and reserves the middle seats of airplanes as unoccupied. With the reduced capacity, we assume 10 apron bus trips each transport 12 passengers from the airport terminal to the airplane. After leaving the bus, in a random sequence, six of the passengers enter the airplane through its front door and the other six through its back door.

We adapted classical boarding methods to accommodate this social distancing context. Each adapted method assigns each passenger to an apron bus trip based on the passenger’s airplane seat location. We implemented and tested nine adaptations of the methods under the same initial conditions through the use of an agent-based model in NetLogo. We conduct experiments when there is 1 m aisle social distancing between passengers walking and standing in the aisle and also with 2 m aisle social distancing.

We evaluate the boarding methods through four performance metrics. Three of the metrics are related to the risk of virus spread. The total number of seat interferences refers to the number of times a passenger needs to leave his or her seat to clear the way for a later boarding window seat passenger to sit down. The other two health-related metrics refer to the risk from passengers walking by previously seated passengers. These metrics are the aisle seat risk duration and window seat risk duration—and depend upon whether the seated passenger passed has an aisle or window seat. Because the aisle seats are closer to the aisle, the aisle seat risk is more important than the window seat risk. The fourth performance metric is the average time to complete boarding of the airplane.

According to our stochastic simulations, the adapted Back-to-front method has the lowest window seat risk, but this method’s performance is poor for the other three metrics and thus is a bad choice for airlines to consider.

The three adapted versions of the Reverse pyramid method have the best health metrics. They have zero seat interferences, the lowest value of aisle seat interferences, and perform better on window seat risk than the other methods excluding the adapted Back-to-front method. The Reverse pyramid – Gradual method has the lowest window seat risk, followed by the Reverse pyramid – Steep method, followed by the Reverse pyramid – Spread method.

The Reverse pyramid – Spread method has the shortest boarding times, followed closely by Reverse pyramid – Steep, which is 12 seconds slower for the high volume luggage situation (S1) with 1 m aisle social distancing in which the former method’s advantage is greatest. The Reverse pyramid – Gradual method is 22 seconds slower than the Reverse pyramid – Steep method for that test condition.

If an airline considers boarding time important and window seat risk unimportant, then the Reverse pyramid – Spread method would be a good choice. If airline considers window seat risk important, then the Reverse pyramid – Gradual method would be a good choice. If an airline considers boarding time important and window seat risk significant, then it may prefer the Reverse pyramid – Steep method.

While we examined three metrics related to passenger health, we did not map the relationship between these metrics to the probabilities of infectious spread. We leave this mapping for infectious disease specialists.

Other future research may vary the number of apron bus trips, the socially distanced capacities of the buses, and the configuration of the airplane. For example, we considered only a single-aisle single-cabin airplane with two doors using apron buses (rather than jet bridges leading to a one-door airplane).

Consistent with [18], we observe that the risk of infection spread to previously seated passengers decreases when the aisle social distance increases from 1 m to 2 m. A generalization of this observation is that when one person is following another, increasing the social distance between the two will reduce the health risk to nearby people they pass. Perhaps future researchers will apply this insight and their creativity to pandemic-related contexts that do not involve airplane boarding.

The paper is accompanied by a series of videos made for S1 luggage situation, for all the considered methods, for both 1 m and 2 m aisle social distancing. The videos can be accessed at the following link: https://github.com/liviucotfas/ieee-access-airplane-boarding-covid19-apron.
